# In Vitro–In Silico Approach in the Development of Clopidogrel Solid Dispersion Formulations

**DOI:** 10.3390/bioengineering12040357

**Published:** 2025-03-30

**Authors:** Ehlimana Osmanović Omerdić, Sandra Cvijić, Jelisaveta Ignjatović, Branka Ivković, Dragana Vasiljević

**Affiliations:** 1 Department of Pharmaceutical Technology and Cosmetology, Faculty of Pharmacy, University of Belgrade, 11000 Belgrade, Serbia; sandra.cvijic@pharmacy.bg.ac.rs (S.C.); dragana.vasiljevic@pharmacy.bg.ac.rs (D.V.); 2 Development and Registration Department, Bosnalijek d.d., 71000 Sarajevo, Bosnia and Herzegovina; 3 Department of Global Nonclinical Safety and Drug Metabolism and Pharmacokinetics, Boehringer Ingelheim Pharma GmbH & Co. KG, 88400 Biberach an der Riss, Germany; 4 Department of Pharmaceutical Chemistry, Faculty of Pharmacy, University of Belgrade, 11000 Belgrade, Serbia

**Keywords:** solid dispersions, clopidogrel, poloxamer 407, copovidone, in vitro dissolution test, in silico physiologically based biopharmaceutics modeling

## Abstract

The aim of this study was to investigate the influence of solid dispersion (SD) formulation factors on improvement of the bioavailability and pharmacokinetic profile of clopidogrel after peroral administration using an in vitro–in silico approach. A clopidogrel-specific, physiologically based biopharmaceutical model (PBBM) was developed and validated to predict absorption and distribution of clopidogrel after peroral administration of the tested formulations. Clopidogrel solid dispersions were prepared using two polymers (poloxamer 407 and copovidone) and a drug-to-polymer ratio of 1:5 and 1:9. The results of the in vitro dissolution test under pH–media change conditions showed that the type and ratio of polymers notably influenced the release of clopidogrel from the SDs. It can be observed that an increase in the polymer content in the SDs leads to a decrease in the release of clopidogrel from the SDs. The predictive power of the constructed clopidogrel-specific PBBM was demonstrated by comparing the simulation results with pharmacokinetic data from the literature. The in vitro dissolution data were used as inputs for the PBBM to predict the pharmacokinetic profiles of clopidogrel after the peroral administration of SDs. SDs with copovidone (1:5) and poloxamer (1:9) showed the potential to achieve the highest drug absorption and bioavailability, with an improvement of over 100% compared to an immediate-release (IR) tablet. The sample with poloxamer (1:9) may have the potential to reduce inter-individual variability in clopidogrel pharmacokinetics due to absorption in the cecum and colon and associated lower first-pass metabolism in the liver. This suggests that distal intestine may be the targeted delivery site for clopidogrel, leading to improved absorption and bioavailability of the drug. This study has shown that an in vitro–in silico approach could be a useful tool for the development and optimization of clopidogrel formulations, helping in decision making regarding the composition of the formulation to achieve the desired pharmacokinetic profile.

## 1. Introduction

Antiplatelet drugs are among the most commonly prescribed therapies, as ischemic heart disease and stroke are the leading causes of death worldwide, accounting for 27% of all deaths worldwide [1,2]. Clopidogrel is one of the most commonly used antiplatelet drugs. In 2020, it was listed as the most commonly prescribed antiplatelet drug in the United States [3]. It has demonstrated efficacy in reducing the incidence of stroke, ischemic attack, myocardial infarction, and unstable angina in patients with atherosclerotic cardiovascular disease [4,5]. Clopidogrel acts by selectively and irreversibly inhibiting the binding of adenosine diphosphate to its platelet receptor, thereby preventing platelet aggregation and clot formation [4]. Typically, clopidogrel is administered as a loading dose of either 300 mg or 600 mg, depending on the established disease, and treatment should be continued with a 75 mg single dose of clopidogrel [4].

Clopidogrel is a pro-drug; 85–90% of the administered dose undergoes extensive first-pass metabolism in the liver [6,7], where it is hydrolyzed to an inactive metabolite (a carboxylic acid metabolite known as SR26334). The remaining 10–15% of the dose undergoes two oxidative reactions involving multiple cytochrome P450 enzymes [8]. Only about 2% of the administered dose is converted to an active metabolite and reaches systemic circulation [6]. Despite its proven safety and efficacy, a significant percentage (5–40%) of patients treated with clopidogrel do not receive the expected therapeutic benefit, and clopidogrel administration has been associated with an increased risk of adverse effects. Genetic polymorphism, drug resistance, platelet reactivity, poor drug solubility, and highly variable clopidogrel release kinetics under changing gastrointestinal conditions may explain the poor response to clopidogrel therapy and inter-individual variations [9,10,11]. Clopidogrel hydrogen sulfate is a class II (low solubility, high permeability) drug according to the Biopharmaceutics classification system (BCS), with pH-dependent aqueous solubility (it is practically insoluble in water at neutral pH but freely soluble at pH 1) [4,12].

The formulation of solid dispersions is one of the most successful strategies for improving the solubility and bioavailability of poorly soluble drugs. The principle of the solid dispersion approach is to disperse a poorly soluble drug in an appropriately selected hydrophilic polymer. The behavior of solid dispersions in the physiological environment depends on the properties of the drug, formulation factors, and physiological conditions in the gastrointestinal tract (GIT) [13,14].

When administered orally, a drug must first be released from the dosage form and dissolved in the gastrointestinal fluids before it can penetrate biological membranes and enter systemic circulation. The drug release mechanism can be explained by using different mathematical models such as zero-order, first-order, Korsmeyer–Peppas, Higuchi, and Hixson–Crowell models based on experimental data [15,16]. The purpose of modeling is to understand the mechanisms of drug release from different formulations. The main release mechanisms for polymer-based formulation are diffusion, polymeric matrix swelling, and polymeric degradation/erosion mechanisms [17]. For peroral solid dosage forms, understanding drug dissolution is critical to identifying which aspects of the formulation and manufacturing process influence the in vitro release rate. Developing in vitro dissolution method requires the selection of suitable experimental conditions that closely simulate the conditions in the GIT [18]. The use of media that simulate pH changes in the GIT could be a very useful dissolution approach due to the notable influence of pH on solubility, especially for weakly basic and acidic drugs.

Physiologically based biopharmaceutics modeling (PBBM) is a valuable tool for the mechanistic interpretation and prediction of drug absorption and distribution, as it mimics physiological conditions and accounts for drug properties and dissolution information to predict a drug’s absorption [19,20,21]. PBBM emphasizes the link between the physicochemical properties of the drug, the formulation characteristics of the dosage form, and the specific physiological conditions in order to understand the impact of these factors on the dissolution, absorption, and distribution of the drug. PBBM can be used at various stages of drug development, including formulation development and selection, the prediction of dosage form bioperformance, the development of biopredictive dissolution methods, the assessment of food effects, and the prediction of drug–drug interactions [21,22].

The aim of this study was to investigate the influence of solid dispersion formulation factors (polymer type and drug-to-polymer ratio) on the improvement of the bioavailability and pharmacokinetic profile of clopidogrel after peroral administration using an in vitro–in silico approach. To predict the absorption and distribution of clopidogrel after peroral administration of the investigated solid dispersions, a clopidogrel-specific, physiologically based biopharmaceutical model had to be developed and validated. In addition, this work aimed to demonstrate the importance of in vitro–in silico approaches in the development of drug dosage forms with target quality characteristics.

## 2. Materials and Methods

### 2.1. Materials

Clopidogrel hydrogen sulfate (CHS) (Ph. Eur.) was used as the active pharmaceutical ingredient. Poloxamer 407 (Kolliphor^®^ P407, BASF, Ludwigshafen, Germany) and copovidone (Kollidon^®^ VA 64, BASF, Ludwigshafen, Germany) were used as hydrophilic polymers. Ethanol (96% *v*/*v*) was the solvent for the preparation of the SDs. All excipients were of pharmaceutical grade.

Hydrochloric acid (30% *v*/*v*), acetic acid, sodium acetate, potassium dihydrogen phosphate, and sodium hydroxide (all manufactured by Merck KGaA, Darmstadt, Germany) were used to prepare the media for CHS solubility and in vitro dissolution studies.

The reagents used for the HPLC analysis were of analytical grade and have been described in our previous work [23].

### 2.2. Methods

#### 2.2.1. Determination of Clopidogrel Hydrogen Sulfate Solubility

The solubility of CHS was tested in hydrochloric acid (pH 1.2), acetate buffer (pH 4.5), and phosphate buffer (pH 6.8). An excess amount of CHS was dispersed in 25 mL of each medium, and the suspensions were shaken for 24 h at 100 rpm and 37 ± 0.5 °C (LSB Aqua Pro Shaking Bath, Grant Instruments, Royston, UK). The samples were filtered using a 0.45 µm filter (Merck Millipore, Merck KGaA, St. Louis, MO, USA). Clopidogrel content was analyzed using the validated RP-HPLC method [24]. All analyzes were performed in triplicate, and the results were expressed as mean ± standard deviation.

When the solubility of CHS in media of different pH values was determined, the dose number (D_0_) was calculated using the following equation:

(1) $$D_{0}=\frac{D}{V_{0}\times C_{0}}$$

where D is the administered dose (mg), V_0_ is the volume of water (250 mL), and C_0_ is the maximum aqueous solubility (mg/mL).

A drug can be considered highly soluble if the dose number is less than 1 and poorly soluble if the dose number is greater than 1 [25]. The dose number was calculated for 75 mg, 300 mg, and 600 mg of usual clopidogrel therapeutic doses.

#### 2.2.2. Preparation of Solid Dispersions

Two different hydrophilic polymers, poloxamer 407 (P) and copovidone (C), were used to prepare the CHS solid dispersions. The drug-to-polymer ratios were 1:5 (sample designations P5 and C5) and 1:9 (sample designations P9 and C9). The polymers copovidone and poloxamer 407 and the drug-to-polymer ratio (1:5 and 1:9) were selected based on the results of our previous research [24]. The solid dispersions were prepared by the solvent evaporation method described in the previous publication [24]. The prepared SDs were filled into hard gelatin capsules (size 00), each capsule containing 75 mg of clopidogrel. The samples were stored in a sealed amber glass bottle with a polyethylene screw under controlled room conditions (22 °C ± 2 °C, 55% RH ± 10% RH) until analyses were performed.

#### 2.2.3. Dissolution Studies

In vitro dissolution studies of clopidogrel SDs, filled in hard gelatin capsules, were performed in a reciprocating cylinder apparatus (Bio-Dis Extended Release Tester, VanKel Technology Group, Cary, NC, USA). Experimental conditions were set to simulate transport of dosage form through different parts of the GIT under fasting conditions (Table 1). Aliquots of 5 mL were withdrawn at predetermined time points and replaced with an equal volume of dissolution medium. The samples were filtered through a 0.45 µm filter (Merck Millipore, Merck KGaA, St. Louis, MO, USA) into HPLC vials. Clopidogrel content was determined using the validated RP-HPLC method, as described in the previously published work [23]. In vitro dissolution studies were performed in triplicate, and data were expressed as mean ± standard deviation.

To explain the mechanism of clopidogrel release from SDs, the obtained dissolution profiles were fitted to different mathematical models (zero-order, first-order, Korsmeyer–Peppas, Higuchi, and Hixson–Crowell models), and the corresponding correlation coefficients (R^2^) were calculated [15,16].

#### 2.2.4. Clopidogrel-Specific PBBM Model

GastroPlus™ software (version 9.8.2000, Simulations Plus Inc., Research Triangle Park, NC, USA) was used to predict the pharmacokinetics of clopidogrel and the influence of formulation factors (in terms of drug release rate from the formulations with different polymer type and drug-to-polymer ratio) on the absorption and distribution of clopidogrel after peroral administration of the SDs studied.

Construction of the PBBM model included the selection of input parameters, optimization, and validation. The model was used to estimate the pharmacokinetics of the formulated SDs with CHS. A drug-specific model was developed based on input parameters related to the physicochemical and pharmacokinetic properties of clopidogrel obtained from the literature, determined experimentally, or estimated by GastroPlus™ software. Selected input parameters are summarized in Table 2. The Advanced Compartmental Absorption and Transit (ACAT) model of human GIT for healthy adults (70 kg) in the fasting state was used to describe the performance of clopidogrel. This model divides the gastrointestinal tract into nine compartments: stomach, duodenum, two jejunum compartments, three ileum compartments, cecum, and ascending colon [26]. Each segment is defined by its physiological properties (length, radius, pH, volume, transit time). The physiological properties were chosen as the default software parameters, with the exception of percent fluid volume in the small intestine (23%) and colon (0.5%) [27,28]. A GastroPlus™ PKPlus™ module was used to estimate the pharmacokinetics of clopidogrel using a three-compartment model based on plasma concentration profiles from intravenous (i.v.) data for various clopidogrel doses [29]. Drug dissolution rate for immediate-release (IR) tablets was estimated using the software default Johnson equation [30]. Human intestinal permeability was estimated using GastroPlus™ software integrated permeability converter based on Caco-2 cell data from literature [31]. Digital extraction of published data from the literature was performed using WebPlotDigitizer v4.4 software (Automeris LLC, Dublin, CA, USA). The experimentally determined solubility values were used in the model as tabulated data across different pH values with interpolation between pH values. The PBBM model was validated by comparing the simulation results with published data from the in vivo studies [8,29]. The fold error between the predicted and observed pharmacokinetic parameters (C_max_, t_max_, AUC_0→∞_, AUC_0→t_) was calculated as follows [32,33]:

(2) $$Fold\;error=\frac{predicted}{observed}$$

**Table 2:** Summary of input parameters for clopidogrel-specific PBBM model.

Parameter	Value
Molecular weight	321.82
log D (pH 7.4)	3.9 ^a^
pK_a_ value (base)	4.55 ^b^
Solubility at 37 °C (mg/mL)	see Table 3 ^c^
Human effective permeability, P_eff_ (cm/s)	4.7767 × 10^−4 d^
Diffusion coefficient (cm^2^/s)	0.7397 × 10^−5 e^
Drug particle diameter (µm)	150 (D_50_); 250 (D_90_) ^f^
Drug dose (mg)	1, 10, 100, 300 (i.v.) ^g^; 75, 300 (p.o.) ^h^
Volume of fluid taken with drug (mL)	200 ^h^
Plasma fraction unbound (%)	2 ^i^
Blood/plasma concentration ratio	0.72 ^b^
First-pass effect in the liver, (FPE %)	96.5 ^j^
Clearance, C_L_ (L/h/kg)	1.2 ^k^
Volume of distribution, V_d_ (L/kg)	0.073 ^k^
Distribution rate constant, k_12_ (1/h)	9.285 ^k^
Distribution rate constant, k_21_ (L1/h)	2.058 ^k^
Distribution rate constant, k_13_ (1/h)	1.243 ^k^
Distribution rate constant, k_31_ (1/h)	0.17 ^k^
Elimination half-life, t_1/2_ (h)	4.4 ^l^

^a^ Literature value taken from [34]. ^b^ Literature value taken from [31]. ^c^ Experimental values. ^d^ Calculated using GastroPlus™ software integrated permeability converter based on Caco-2 cell data from [31]. ^e^ GastroPlus™ software calculated (based on drug molecular weight). ^f^ Certificate of analysis data taken from the drug manufacturer. ^g^ Literature values taken from [29]. ^h^ Literature values taken from [8]. ^i^ Literature value taken from [35]. ^j^ Optimized to match simulated to the in vivo plasma concentration profile in accordance with literature data [6]. ^k^ Calculated using GastroPlus™ PKPlus™ module based on the i.v. data [29]. ^l^ GastroPlus™ software calculated.

**Table 3:** Results of solubility test and dose number calculations.

pH Value	Solubility (mg/mL) ± S.D.	Therapeutic Dose (mg)		
		75	300	600
		Dose Number		
1.2	268.750 ± 6.159	0.001	0.004	0.009
4.5	0.055 ± 0.005	5.455	21.818	43.636
6.8	0.016 ± 0.003	21.429	85.714	171.429

S.D.—standard deviation.

The prediction was considered acceptable if the predicted values were within two-fold range of the observed values [32,33]. The developed model can be used to simulate the pharmacokinetics of patients with cardiovascular disease, but the numerous physiological differences, such as altered hepatic blood flow, reduced gastrointestinal motility, protein binding, volume of distribution, and drug clearance, must be taken into account when adapting the model for this population.

Using the experimental dissolution data obtained under pH–media change conditions as additional inputs, the clopidogrel-specific PBBM model was used to estimate drug absorption and distribution after peroral administration of the studied SD formulations. The dissolution profiles were entered directly into the software using the “CR: Dispersed” formulation option with .dsd files.

## 3. Results and Discussion

### 3.1. Determination of Clopidogrel Hydrogen Sulfate Solubility

The results of the solubility test of CHS (Table 3) confirm the pH-dependent solubility of this drug [4,12]. CHS was freely soluble at pH 1.2 (268.750 ± 6.159 mg/mL). The solubility of the drug was markedly lower at higher pH values (0.055 ± 0.005 mg/mL at pH 4.5 and 0.016 ± 0.003 mg/mL at pH 6.8). The observed solubility was consistent with expectations based on the weakly basic nature of clopidogrel. The calculated dose number for therapeutic doses (75 mg, 300 mg, and 600 mg) was less than 1 at pH 1.2, while it was greater than 1 at pH 4.5 and 6.8 (Table 3). It can be concluded from the results that pH-dependent solubility is a limiting factor for the absorption of clopidogrel.

### 3.2. Dissolution Studies

**Figure 1:**
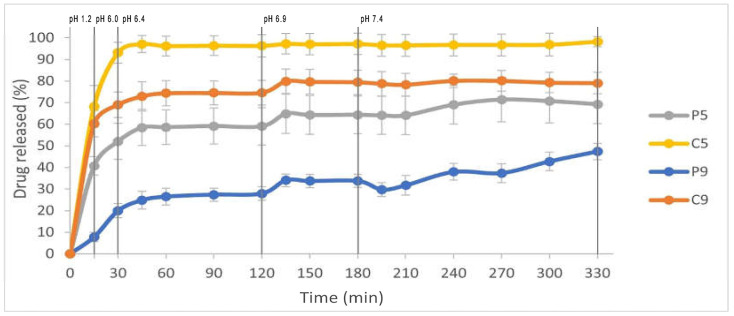
Comparative dissolution profiles of clopidogrel solid dispersions.

The prepared solid dispersions were used for a dissolution study under pH–media change conditions. The dissolution profiles of clopidogrel from SDs are shown in Figure 1. A rapid and almost complete release of clopidogrel was achieved from sample C5, with 96.21% of the drug released within 60 min of testing. The amount of clopidogrel released from C9 and P5 after 60 min was lower, reaching 74.38% and 58.65%, respectively. For these three formulations (C5, C9, and P5), it can be observed that a plateau in the dissolution curve was achieved for C5 at approximately 60 min and for C9 and P5 at around 135 min of dissolution testing. Sample P9 released the smallest amount of clopidogrel, with only 26.48% of the drug dissolved after 60 min. It can be observed that an increase in the polymer content (poloxamer 407 or copovidone) led to a decrease in clopidogrel release from the SDs. This could be explained by the assumption that the polymers in the SDs start to swell in the medium and form a viscous polymer layer around the drug particles, which slows down the dissolution of clopidogrel [36,37,38]. In addition, the high polymer content in SDs leads to slow dissolution due to intermolecular interactions between the drug and polymer, resulting in reduced drug molecular mobility [39,40]. These results are in contrast to our previously published study [24], where an increase in the amount of the hydrophilic polymer in SDs (poloxamer 407, copovidone, povidone, or macrogol 6000) was associated with an increase in clopidogrel dissolution when phosphate buffer pH 6.8 was used as the medium for the dissolution test. It can therefore be concluded that the conditions under which the dissolution test is performed have a noticeable effect on the release of clopidogrel from SDs. The volume of the medium can also influence the release rate of the drug from poloxamer-based formulations. This is because the poloxamer gel that forms around the drug particles tends to degrade faster in larger volumes of the dissolution medium [41]. In the earlier study [24], a volume of 900 mL of dissolution medium was used, and the P9 formulation achieved the highest and almost complete release of clopidogrel compared to the other formulations. In this study, a volume of 240 mL was used for each pH medium, and clopidogrel release was the slowest from sample P9.

The results of the in vitro dissolution study under conditions of pH–media change are consistent with the findings on the solubility of CHS, where the highest solubility of CHS was observed in a medium with a pH of 1.2. Among the three solid dispersions (C5, P5, and C9), clopidogrel showed a swift onset of dissolution in the pH 1.2 medium, as indicated by a steep increase in the dissolution curve (Figure 1). The dissolution of clopidogrel slowed down during the media change (from pH 6.0 to 6.4), with a plateau being reached for C5 after 60 min at pH 6.4 and for P5 and C9 after 135 min at pH 6.9. It can be observed that clopidogrel was released slowly and gradually from sample P9, with the maximum release (47.37%) being reached at the end of the test after 330 min. The obtained results demonstrate that the proper selection of polymer type and ratio, as well as the dissolution conditions, are crucial for the release of clopidogrel from SDs.

Among the tested SDs, the Huguchi model best described the release kinetics of clopidogrel from samples C5, P5, and C9, as shown by the highest R^2^ value (Table 4). The release process from the mentioned formulations was controlled by the diffusion of the drug through the pores of the polymer matrix. The model is based on Fick’s first law of diffusion, which states that the diffusion rate is proportional to the concentration gradient, i.e., the concentration gradient decreases as the drug is released from the matrix [15]. On the other hand, the Korsmeyer–Peppas model provided the best description of the release kinetics of clopidogrel from the P9 formulation, as shown by the highest R^2^ value calculated for this model (Table 4). The Korsmeyer–Peppas mathematical model is based on the assumption that drug release occurs through a combination of Fick’s diffusion and polymer erosion. The diffusional exponent (n) for P9 was 1.83, higher than the value of 0.89, indicating that the release of clopidogrel from this SD occurred mainly by the mechanism of polymer erosion [42]. It can be observed that formulations P5 and P9, prepared with the same polymer (poloxamer 407) but with different content (drug-to-polymer ratios of 1:5 and 1:9, respectively), exhibited different mechanisms of clopidogrel release kinetics. The poloxamer gel matrix formed porous structures in contact with the medium, which could be the reason why diffusion was the predominant mechanism for the release of the drug from sample P5. In the case of P9, increasing the amount of polymer promoted gel strength, reduced pores, and slowed down the release of the drug with the predominant release mechanism of polymer erosion [41].

### 3.3. PBBM Model Construction and Validation

**Figure 2:**
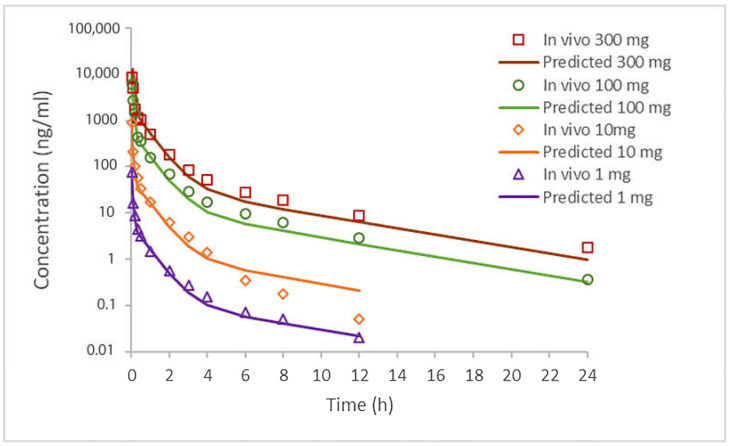
Predicted and observed (mean in vivo) pharmacokinetic profiles of clopidogrel after i.v. administration of 1 mg, 10 mg, 100 mg, and 300 mg doses.

**Figure 3:**
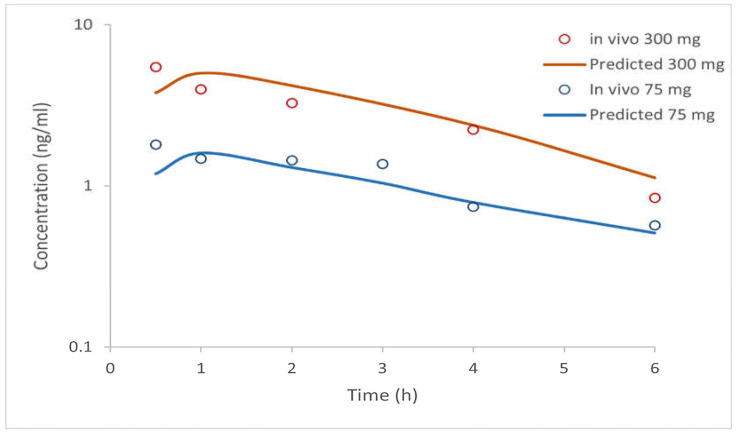
Predicted and observed (mean in vivo) pharmacokinetic profile of clopidogrel after p.o. administration of 75 mg and 300 mg doses.

Based on selected input data (Table 2), the simulation results for i.v. administered doses of clopidogrel (1 mg, 10 mg, 100 mg, and 300 mg) and p.o. administered doses as IR tablets (75 mg and 300 mg) are shown in Figure 2 and Figure 3 and in Table 5 and Table 6. The software-predicted clopidogrel elimination half-life (t_1/2_) of 4.4 h was within the range of values from published data (3.94–5.69 h) [43,44], demonstrating that selected model parameters appropriately describe the pharmacokinetics of clopidogrel. All tested intravenous doses resulted in simulated AUC_0→∞_ and AUC_0→t_ values that were within a two-fold error of the mean in vivo data (Table 5), indicating that the simulated profiles well describe the pharmacokinetics of clopidogrel following i.v. administration. In order to further validate the generated model, drug pharmacokinetics were predicted for clopidogrel 75 mg and 300 mg IR tablets under fasting conditions. The predicted pharmacokinetic parameters (C_max_, t_max_, AUC_0→∞_, and AUC_0→t_) (Table 6), falling within a two-fold range, are in good agreement with the mean in vivo reported data [8]. The PBBM model was developed using data from healthy subjects and used for simulations in patients with cardiovascular disease. Based on previous considerations, it can be concluded that the constructed model properly describes the pharmacokinetics of clopidogrel and can be used to predict the influence of formulation factors on drug absorption and distribution.

### 3.4. PBBM Model Exploration

To evaluate the impact of formulation factors on clopidogrel absorption, the results of in vitro dissolution studies under conditions of pH–media change for four SD formulations (P5, C5, P9, and C9) were used as input data in the in silico model (Figure 1). Drug dissolution profile from IR tablets were generated by the software integrated equation. The simulated PK parameters for IR tablet and SDs (containing 75 mg dose) are shown in Table 7.

If the solubility in the stomach exceeds that in the small intestine, precipitation of the drug may occur in the gastrointestinal tract. Although precipitation was considered in the PBBM model, the concentration–time profiles for the solid dispersions showed no decrease in dissolved drug concentration after the initial increase, showing that precipitation did not occur. This is likely due to the presence of the polymers (copovidone and poloxamer 407) in large amounts (drug-to-polymer ratios of 1:5 and 1:9), which act as precipitation inhibitors and help to stabilize the dissolved drug [45,46].

The mean C_max_ of unchanged clopidogrel after a single peroral dose of 75 mg was approximately 2.2–2.5 ng/mL [4], and this agrees well with the simulated C_max_ for the IR tablet based on the software-calculated dissolution (2.0 ng/mL). The simulated C_max_ values for the SDs were notably higher than for the IR tablet (Table 7). Karaźniewicz-Łada et al. [8] reported that the C_max_ value measured in vivo after administration of 300 mg of clopidogrel in the form of IR tablets was 5.47 ng/mL (Table 6). These values are notably lower than the predicted C_max_ for SDs with 75 mg clopidogrel. The simulation results also indicate that increased absorption, bioavailability, and overall exposure of the drug (as indicated by higher AUC values) can be expected for all solid dispersions studied. This is evidenced by an increase in percentage of drug dose absorbed (F_a_), bioavailability (F_b_) and AUC values, with the expected improvement compared to IR tablets ranging from more than 50% to 120%, depending on the formulation of the SDs. The highest percentage of F_a_, F_b_, and AUC can be expected for formulations C5 and P9, with improvements of more than 100% for F_a_ and F_b_ and 72.22% and 61.11%, respectively, for AUC compared to the IR tablet. These results suggest that the use of solid dispersions could allow for a reduction in the therapeutic dose of clopidogrel while ensuring a satisfactory therapeutic response.

**Figure 4:**
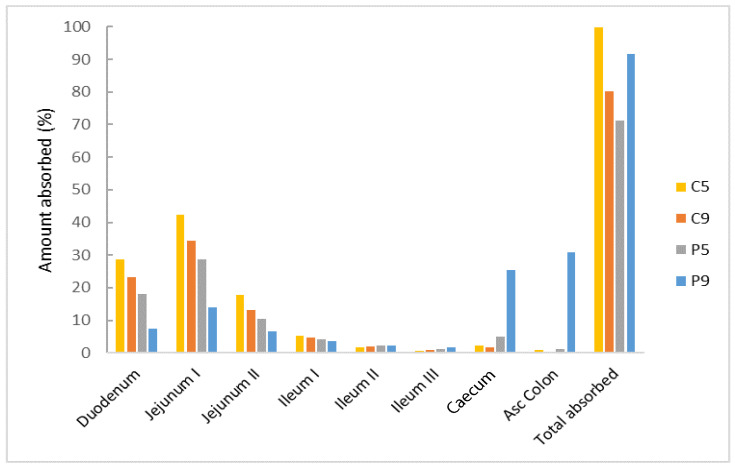
Simulation of regional clopidogrel absorption distribution.

The simulation of the regional absorption distribution presented in Figure 4 shows that the majority of clopidogrel released from C5, P5, and C9 is expected to be absorbed in the duodenum and jejunum (88.8%, 57.2%, and 70.7% of the total absorbed dose, respectively), while a smaller fraction will be absorbed in the distal part of the intestine and colon. For the P9 formulation, clopidogrel absorption is expected throughout the entire intestine, with dominant absorption in the cecum and colon (56% of the total absorbed dose), due to prolonged drug release.

The results of the simulations suggest that the largest amount of clopidogrel, almost 100% of the dose, will be absorbed after release from the C5 formulation. These findings follow the observed trend in the in vitro dissolution test, which revealed the fastest and highest release of clopidogrel from the C5 formulation. The relatively high predicted value of F_b_ for P9, as a result of absorption in the lower parts of the GIT, can also be explained by the assumption that the drug dose absorbed in these parts probably does not undergo first-pass metabolism in the liver. Due to absorption in the lowest part of the intestine and, thus possibly, reduced first-pass metabolism, formulation P9 is expected to have the lowest drug concentration in the liver (C_max_ liver of 18 ng/mL; Table 7) compared to other SDs tested. These results suggest that the P9 formulation, among the other SDs tested, could potentially contribute to a reduction in inter-individual variation in therapy.

Based on these simulations, it can be suggested that the cecum and colon could be targeted delivery sites for clopidogrel, which could lead to improved absorption and bioavailability of the drug. Attempts to achieve a modified release of clopidogrel to improve therapeutic response and reduce adverse effects have already been documented in the literature [47,48,49]. However, this is the first time that an in vitro–in silico approach has been used in the development and optimization of clopidogrel solid dispersion formulations.

The results emphasize the importance of careful selection of the polymer type and concentration in SDs, which could contribute to improved biopharmaceutical properties of clopidogrel SDs.

## 4. Conclusions

In this study, a validated clopidogrel-specific PBBM model was used to investigate the effects of solid dispersion formulation factors on the absorption and distribution of clopidogrel. Cost-effective manufacturing processes and known safe excipients (copovidone and poloxamer 407) were used to prepare the clopidogrel solid dispersions. In vitro dissolution tests under the condition of pH–media change showed significant differences in the dissolution rates of the different SDs, emphasizing the importance of careful selection and quantification of polymers in the formulation of SDs. The in vitro dissolution method has been shown to successfully simulate pH changes in the GIT and can be used for model predictions of SD formulations.

According to the modeling results, the solid dispersions C5 (drug-to-copovidone ratio 1:5) and P9 (drug-to-poloxamer 407 ratio 1:9) showed the potential to achieve the highest drug absorption and bioavailability, with an improvement of over 100% compared to an IR tablet. In addition, all simulated values of the clopidogrel pharmacokinetic parameters are notably higher than those of the IR tablet. The simulation results indicate that the use of solid dispersions could potentially enable a reduction in the therapeutic dose of clopidogrel and reduce the risk of clopidogrel side effects. Among the SD formulations tested, sample P9 may potentially reduce inter-individual variability in clopidogrel pharmacokinetics due to drug absorption in the cecum and colon and probably lower first-pass metabolism of the drug in the liver. This suggests that distal intestine might be targeted delivery site for clopidogrel, leading to improved absorption and bioavailability of the drug.

Several PBPK models for clopidogrel have been developed, focusing primarily on the effects of clinical and demographic factors, drug–drug interactions, interindividual variations, and enzyme polymorphisms on clopidogrel metabolism [50,51,52,53,54]. However, all of these models have used commercially available tablet formulations, and none have been developed or applied to novel experimental formulations. To our knowledge, this is the first and only PBBM model for clopidogrel developed to investigate the influence of formulation factors on pharmacokinetic parameters, bioavailability, and distribution after oral administration. This model represents a novel in vitro–in silico approach to under-stand how changes in formulation, e.g., polymer type and drug-to-polymer ratio, can affect the pharmacokinetics of clopidogrel.

This study has shown that an in vitro–in silico approach could be a useful tool for the development and optimization of clopidogrel formulations, helping in decision making regarding the composition of the formulation to achieve the desired pharmacokinetic profile.

## Figures and Tables

**Table 1:** Bio-Dis dissolution test conditions.

Row	I	II	III	IV	V
GIT region	stomach	duodenum	proximal jejunum	distal jejunum	distal ileum
pH value	pH 1.2	pH 6.0	pH 6.4	pH 6.9	pH 7.4
Residence time (min)	15	15	90	60	150
Volume of dissolution medium (mL)	240				
Hold dip time (s)	30				
Drain time (s)	60				

**Table 4:** Calculated correlation coefficient (R^2^) for different mathematical models which describe the release kinetics of clopidogrel from the solid dispersions.

SDs	Zero- Order	First- Order	Korsmeyer–Peppas	Higuchi	Hixon– Crowell
	R^2^	R^2^	R^2^	R^2^	R^2^
P5	0.7549	0.8307	0.9742	0.9769	0.8060
C5	0.7141	0.8787	0.9595	0.9734	0.8422
P9	0.9304	0.9409	0.9937	0.7395	0.9376
C9	0.6603	0.7728	0.9901	0.9984	0.7328

**Table 5:** Predicted and observed [29] pharmacokinetic parameters after i.v. administration of different clopidogrel doses.

	Parameter	AUC_0→∞_ (ng h/mL)	AUC_0→t_ (ng h/mL)
1 mg i.v. injection	Predicted	11.45	11.31
	In vivo mean	10.80	10.71
	Fold error	1.06	1.06
10 mg i.v. injection	Predicted	114.45	113.08
	In vivo mean	121.41	121.25
	Fold error	0.94	0.93
100 mg i.v. infusion	Predicted	1144.50	1142.40
	In vivo mean	1137.60	1135.40
	Fold error	1.01	1.01
300 mg i.v. infusion	Predicted	3433.50	3427.20
	In vivo mean	2406.50	2393.30
	Fold error	1.43	1.43

**Table 6:** Predicted and observed [8] pharmacokinetic parameters after p.o. administration of different clopidogrel doses.

	Parameter	C_max_(ng/mL)	t_max_ (h)	AUC_0→∞_ (ng h/mL)	AUC_0→t_ (ng h/mL)
75 mg IR tablet	Predicted	1.60	0.94	9.43	5.76
	In vivo mean	1.81	0.50	10.87	6.50
	Fold error	0.88	1.88	0.87	0.89
300 mg IR tablet	Predicted	5.03	0.92	21.32	17.69
	In vivo mean	5.47	0.50	17.70	15.95
	Fold error	0.92	1.84	1.20	1.11

**Table 7:** Predicted pharmacokinetic parameters of clopidogrel for IR tablet and solid dispersions (75 mg dose).

Parameter	IR Tablet ^1^	P5 ^2^	C5 ^2^	P9 ^2^	C9 ^2^
F_a_ (%)	45.385	71.330	99.883	91.637	80.126
F_b_ (%)	1.585	2.497	3.496	3.207	2.804
C_max_ (ng/mL)	2.00	12.00	19.00	6.00	15.00
t_max_ (h)	0.96	0.80	0.56	0.80	0.50
AUC_0→∞_ (ng h/mL)	18.00	22.00	31.00	29.00	25.00
AUC_0→t_ (ng h/mL)	14.00	22.00	31.00	28.00	25.00
C_max_ liver (ng/mL)	5.00	35.00	55.00	18.00	42.00

Fa—percent of drug dose absorbed (entered into the enterocytes). Fb—drug bioavailability. ^1^ Predicted data based on dissolution profile calculated using software default Johnson equation [30]. ^2^ Predicted data based on experimental in vitro dissolution profile.

## Data Availability

The original contributions presented in the study are included in the article; further inquiries can be directed to the corresponding author.
